# Retinal Imaging as a Window into Cardiovascular Health: Towards Harnessing Retinal Analytics for Precision Cardiovascular Medicine

**DOI:** 10.3390/jcdd12060230

**Published:** 2025-06-17

**Authors:** Jay Bharatsingh Bisen, Hayden Sikora, Anushree Aneja, Sanjiv J. Shah, Rukhsana G. Mirza

**Affiliations:** 1 Department of Ophthalmology, Northwestern University Feinberg School of Medicine, Chicago, IL 60611, USA; jay.bisen@northwestern.edu (J.B.B.); hayden.sikora@northwestern.edu (H.S.); anushree.aneja@northwestern.edu (A.A.); 2 Division of Cardiology, Department of Medicine, Northwestern University Feinberg School of Medicine, Chicago, IL 60611, USA; sanjiv.shah@northwestern.edu

**Keywords:** retinal imaging, cardiovascular disease, optical coherence tomography, optical coherence tomography angiography, color fundus imaging, precision cardiology, artificial intelligence

## Abstract

Rising morbidity and mortality from cardiovascular disease (CVD) have increased interest in precision and preventive management to reduce long-term sequelae. While retinal imaging has traditionally been recognized for identifying vascular changes in systemic conditions such as hypertension and type 2 diabetes mellitus, a new ophthalmologic field, cardiac-oculomics, has associated retinal biomarker changes with other cardiovascular diseases with retinal manifestations. Several imaging modalities visualize the retina, including color fundus photography (CFP), optical coherence tomography (OCT), and OCT angiography (OCTA), which visualize the retinal surface, the individual retinal layers, and the microvasculature within those layers, respectively. In these modalities, imaging-derived biomarkers can present due to CVD and have been linked to the presence, progression, or risk of developing a range of CVD, including hypertension, carotid artery disease, valvular heart disease, cerebral infarction, atrial fibrillation, and heart failure. Promising artificial intelligence (AI) models have been developed to complement existing risk-prediction tools, but standardization and clinical trials are needed for clinical adoption. Beyond risk estimation, there is growing interest in assessing real-time cardiovascular status to track vascular changes following pharmacotherapy, surgery, or acute decompensation. This review offers an up-to-date assessment of the cardiac-oculomics literature and aims to raise awareness among cardiologists and encourage interdepartmental collaboration.

## 1. Introduction

Cardiovascular disease (CVD) remains a significant cause of morbidity and mortality within the United States and worldwide. Between 2012 and 2018, the total cost of cardiovascular conditions, including heart failure, acute myocardial infarction, cerebrovascular disease, and peripheral vascular disease, has steadily increased [1]. The incidence of these conditions is predicted to rise through the next decade [1], prompting a focus on screening methods and biomarkers for disease prevention in addition to the treatment of symptoms.

Numerous epidemiological studies and clinical trials have already been conducted focusing on modifiable aspects of cardiovascular disease, including the Framingham Heart Study focusing on lifestyle factors such as tobacco use and obesity [2], the Systolic Blood Pressure Intervention Trial amending target values for hypertension management [3], and various clinical trials highlighting the benefits of pharmacological therapy such as statins and GLP-1 receptor agonists for the prevention of adverse cardiac events [4,5]. From these landmark research studies came the development of predictive screening tools, such as the Atherosclerotic Cardiovascular Disease (ASCVD) risk calculator, an algorithm incorporating variables such as age, cholesterol levels, blood pressure, and smoking status to assess a patient’s 10-year risk for ASCVD [6], and the more recent sex-specific, race-free American Heart Association (AHA) Predicting Risk of CVD Events (PREVENT) risk calculator, which provides 10- and 30-year estimates of risk of incident ASCVD and heart failure [7].

Despite the advent of risk scores for the prediction of CVD events, which inform preventive strategies, CVD remains the leading cause of death worldwide, and risk prediction is far from personalized to the individual patient. Therefore, in addition to risk prediction metrics derived from exam findings and lab values, imaging-based biomarkers have been developed to evaluate for subclinical CVD. Examples include carotid intima-media thickness and coronary artery calcium (CAC) scores, which are useful in evaluating the risk of stroke and improving risk stratification for coronary artery disease-related events, respectively [8]. However, these tests require specialized equipment and expertise for their acquisition and interpretation, and in the case of CAC scores, they require computed tomography, which involves exposure to radiation. For these reasons, additional noninvasive, accessible tools that provide clinicians and their patients with real-time assessments of cardiovascular status would be valuable additions to current options.

The retina is one of the few places in the body where the vasculature can be examined routinely and easily in the clinical setting. The retinal vasculature is critical for maintaining retinal health and proper functioning, and it also provides a novel, accessible window into the health of the systemic microvasculature. Millions of retinal exams are performed on adults annually in the U.S. alone, and yet this potential wealth of information is currently discarded once information on clinical retinal disease has been evaluated. Thus, retinal imaging represents a tremendous yet underutilized potential resource for CVD risk assessment.

Cardiac-oculomics, a novel field in ophthalmology, is focused on identifying ocular biomarkers from high-resolution imaging modalities to assess retinal manifestations of systemic disease [9]. Retinal imaging has revealed microvascular abnormalities linked to systemic diseases, enabling a more thorough pathophysiologic understanding of retinal microvasculature dysfunction and its relationship to systemic vascular dysfunction. Chronic systemic vascular issues can predispose the retina to chronic ischemic changes and increase the risk of acute retinal infarctions. These retinal ischemic changes can be quantified, and their correlations to the development of cardiovascular disease are being thoroughly assessed [10].

**Figure 1:**
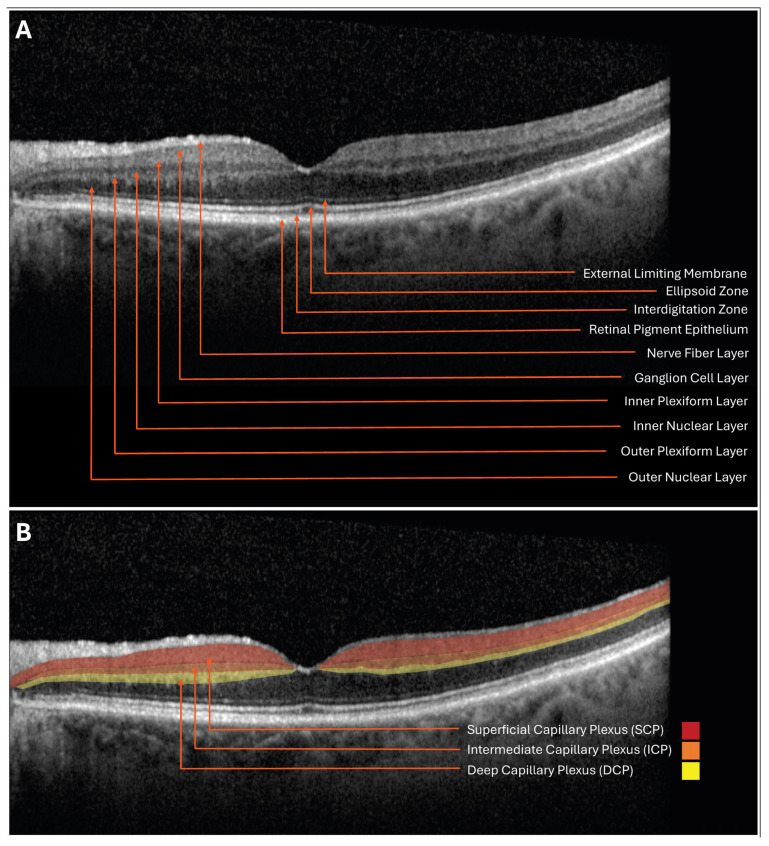
(**A**) Spectral domain optical coherence tomography (SD-OCT) B-scan with retinal layers identified. (**B**) SD-OCT B-scan with retinal capillary plexus layers highlighted.

The retinal ischemic cascade describes a continuum of ischemic events within the retina [Figure 1]. The retinal microvasculature is layered into the superficial, intermediate, and deep capillary plexuses (SCP, ICP, and DCP, respectively), each with unique susceptibilities to ischemia due to differences in blood supply and drainage pathways. Arterial in-flow to these plexuses is localized to the superficial aspect of the retina, while venous out-flow is localized to the posterior aspect of the retina [11]. After the initial ischemic infarction, lesions can progress horizontally or vertically, resulting in varying presentations of ischemia, including more acute, superficial findings such as retinal artery occlusions (RAOs); focal, chronic findings such as cotton wool spots (CWSs); expansive, acute, and deep findings such as paracentral acute middle maculopathy (PAMM); focal, chronic, and deep findings such as retinal ischemic perivascular lesions (RIPLs); acute, deep findings such as retinal vein occlusions (RVOs); and expansive, acute, and deeper findings such as acute macular neuroretinopathy (AMN). Given the vascular and ischemic nature of these findings, they can be identified using high-resolution imaging modalities, and many also confer elevated CVD risk. Ophthalmic imaging holds the potential to be incorporated into clinical workflow as a non-invasive, quick, and low-cost assessment of a patient’s microvasculature and CVD status.

In this review, we explore the retinal imaging modalities available to physicians, the biomarkers derived from these modalities, and how this information can be used to assess cardiovascular status for future implementation in non-ophthalmological settings.

## 2. Retinal Imaging Modalities

**Figure 2:**
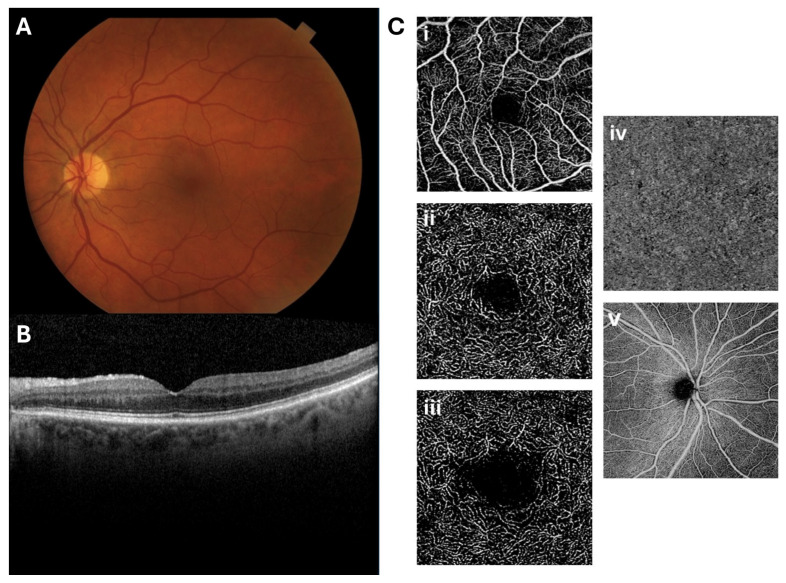
Representative (**A**) color fundus photography (CFP), (**B**) optical coherence tomography (OCT) B-scan, and (**C**) optical coherence tomography angiography (OCTA) en face images. The OCTA images include representative segmentations of the (**i**) superficial capillary plexus (SCP), (**ii**) intermediate capillary plexus (ICP), (**iii**) deep capillary plexus (DCP), (**iv**) choriocapillaris (CC), and (**v**) radial peripapillary capillary plexus (RPCP).

There are various ophthalmic imaging modalities utilized in clinical practice that each have separate indications and strengths. For example, color fundus photography (CFP) provides representations of the retinal surface, while others, such as optical coherence tomography (OCT), have the image resolution to provide additional information of the retina’s various layers and vasculature [Figure 2]. Each of these techniques yields unique information that can be harnessed to better understand the relationship between the ocular and cardiac vascular environments.

### 2.1. Color Fundus Photography

CFP has been used to document clinical exam findings, often in conjunction with other imaging modalities like fluorescein angiography (FA) and indocyanine green angiography (ICGA), invasive ophthalmic modalities requiring specialized equipment and staff to visualize real-time ocular vascular dynamics. Although these imaging techniques are useful in ophthalmology clinics, these modalities are not the focus of this review given their invasive nature and limited wide-scale application outside of the ophthalmologic clinical environment.

CFP captures the fundus, the posterior surface of the eye, which contains the retina, macula, optic disc, fovea, and blood vessels, which has been further optimized as technological innovation has enabled CFP acquisition without dilation [12]. Advanced CFP acquisitions being utilized in retinopathy of prematurity are being developed to harness red, green, and blue (RGB) color channels, with each channel corresponding to a specific wavelength range of light and a specific depth of penetrance that allows different layers and structures of the retina to be more closely examined [13]. Its simplicity allows for use as a basic imaging tool to assess retinal vasculature health.

### 2.2. Optical Coherence Tomography

OCT imaging allows for the evaluation of the retina at a microscopic level. Similar to the theory behind ultrasound imaging, OCT emits light and measures the varied reflected light from the retina, allowing for the creation of a cross-sectional image for visualization, measurement, and detection of morphological alterations of the retina’s distinct layers [14]. OCT captures high-resolution images to quantify aspects of the retina otherwise not visible on color fundus photography but is more expensive and requires greater operator expertise.

### 2.3. Optical Coherence Tomography Angiography

OCT angiography (OCTA) allows for detailed imaging of the retinal microvasculature by capturing sequential OCT scans and quantifying differences in movement between frames, which often is attributable to the movement of red blood cells [15]. This generates an image that maps dynamic blood flow information without the need for contrast [15]. OCTA specifically provides en face images, allowing for visualization of frontal sections of the retina at different capillary plexuses, including the SCP, ICP, DCP, radial peripapillary capillary plexus (RPCP), and choriocapillaris (CC).

These imaging modalities allow clinicians and researchers to employ a multimodal imaging approach to identify retinal diseases and quantify retinal biomarkers with known cardiovascular implications. However, not all retinal findings that confer CVD risk can be quantified. Of these findings, vascular occlusions within the retina, including RAOs and RVOs, have been the most extensively explored given their acuity and significant visual impairment.

## 3. Retinal Diseases Associated with Cardiovascular Risk

### 3.1. Retinal Artery Occlusion

**Figure 3:**
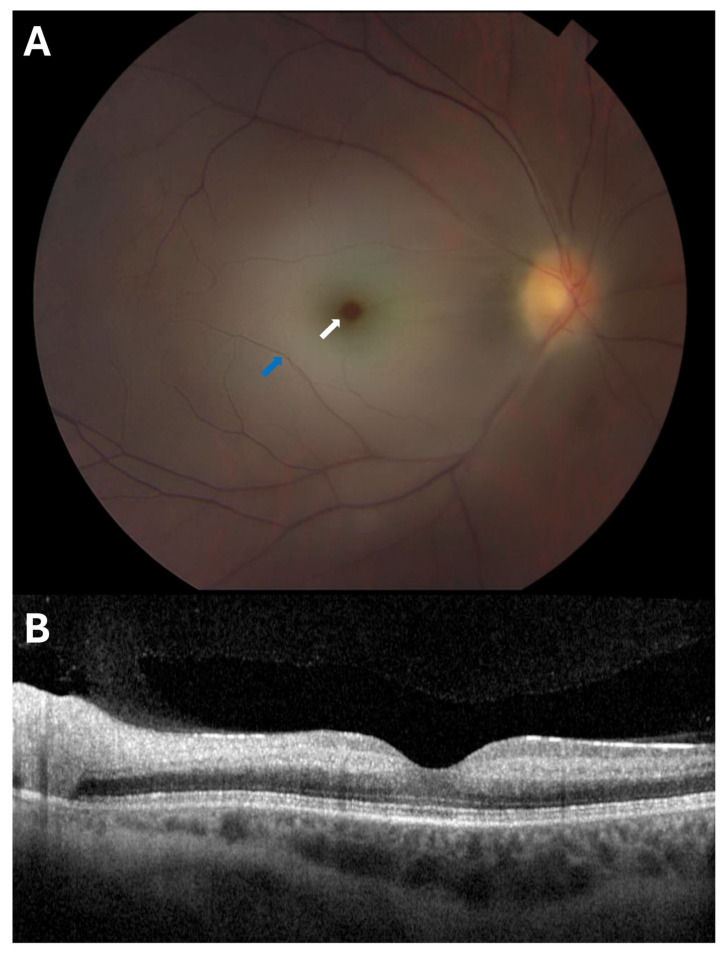
Representative (**A**) color fundus photography (CFP) and (**B**) optical coherence tomography (OCT) B-scan images of an eye with central retinal artery occlusion (CRAO). The “cherry red spot” (white arrow) visible at the center of the CFP image is characteristic of CRAO, as the loss of blood flow brought on by the occlusion causes retinal whitening (blue arrow), while the fovea retains its normal red hue. The OCT image demonstrates the characteristic hyperreflectivity and thickening of the inner retina due to ischemia caused by a CRAO.

Central retinal artery occlusion (CRAO) is the blockage of blood flow from the central retinal artery, the main artery supplying the retina [Figure 3]. Similarly, branch retinal artery occlusion (BRAO) is the blockage of blood flow at a downstream branch of the central retinal artery. RAOs can result from cholesterol, fibrin thrombin, or calcium plaques, but the vast majority, up to 95%, of RAO events are thromboembolic in origin and mechanistically equivalent to an embolic ischemic stroke [16]. Thus, the AHA Stroke Council officially classifies RAO as an ischemic stroke equivalent [17].

Given this classification, RAOs are unsurprisingly strongly associated with increased cardiovascular risk. Of note, studies estimate that around 1/3 of RAO patients have concurrent major carotid disease [18]. Another study found undiagnosed vascular risk factors in 78% of RAO patients, notably ipsilateral carotid artery stenosis (CAS) and arterial hypertension [19]. CRAO has also been associated with increased risk of arrhythmia, specifically atrial fibrillation, with one study finding that CRAO patients were 12 times more likely than the general population to have atrial fibrillation [20]. Additionally, in the first 4 years following an RAO event, a patient has a greater than 25% risk of suffering a myocardial infarction, a stroke, or death [18,20,21,22,23,24].

Together, these associations demonstrate the need for a timely cardiac evaluation following the identification of an RAO event to identify and treat concurrent conditions before presentation.

### 3.2. Retinal Vein Occlusion

**Figure 4:**
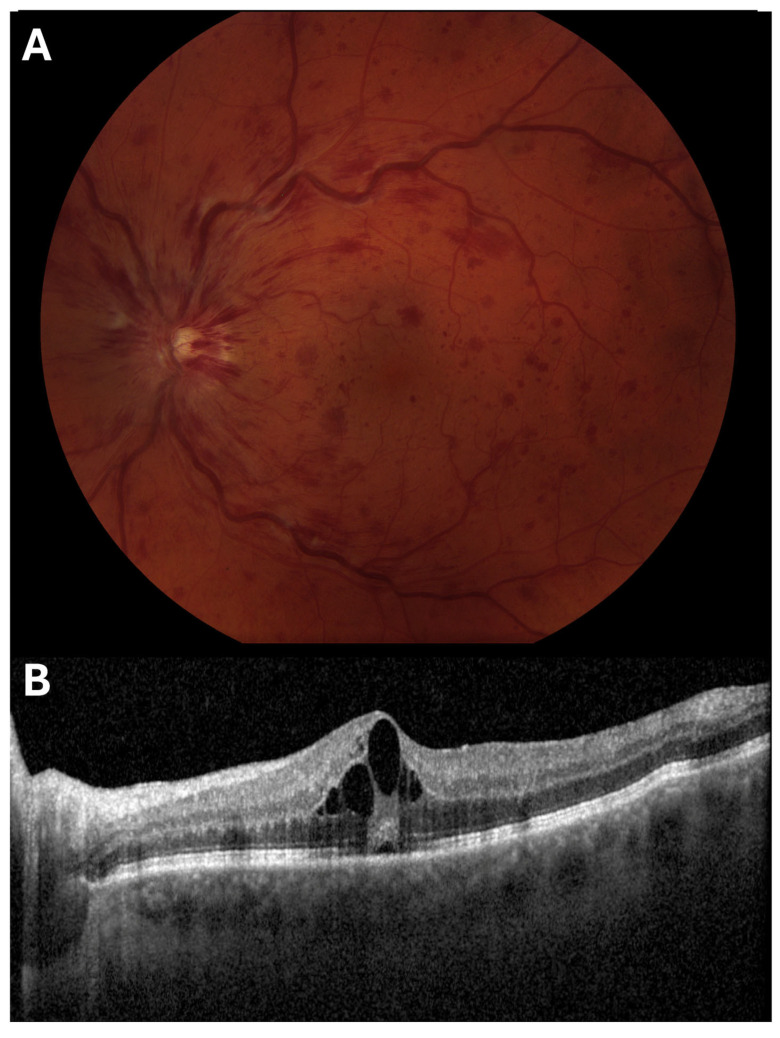
Representative (**A**) color fundus photography (CFP) and (**B**) optical coherence tomography (OCT) B-scan images of an eye with central retinal vein occlusion (CRVO).

Central retinal vein occlusion (CRVO) is the blockage of blood flow through the central retinal vein. Similarly, branch retinal vein occlusion (BRVO) is the blockage of blood flow through a particular branch of the central retinal vein. These occlusions increase retinal capillary pressure, often leading to hemorrhage and macular edema [Figure 4]. While the exact pathogenesis of RVO is unknown, it appears to be preceded by hypercoagulable conditions [10]. Additionally, hypertension, diabetes, and open-angle glaucoma are common risk factors for RVO [25].

In addition to its shared risk factors with cardiovascular disease, several studies have associated RVO with increased cardiovascular risk. CRVO patients have an increased risk of CVD incidence compared to age-matched controls [26] and myocardial infarction [27]. Also, both RVO types are associated with an increased risk of MI, peripheral artery disease, congestive heart failure, and stroke [28,29]. Together, these associated increases in cardiovascular risk demonstrate the importance of diagnosing RVO and potentially initiating statin-oriented treatment for CRVO [30].

As described earlier, these vascular occlusions often present globally across the retina and can be treated similarly to larger adverse cardiac events. However, the retina and its microvasculature are sensitive to subclinical causes of ischemia or hypoperfusion, leading to structural and functional retinal changes. Retinal imaging has shown promise in identifying biomarkers and quantifying vascular changes that present prior to the onset of larger-scale ischemia, allowing clinicians a viewpoint into a patient’s systemic vascular health.

## 4. Overview of the Known Retinal Vasculature Biomarkers

In this section, we will introduce the various biomarkers that can be measured or identified in the CFP [Table 1], OCT [Table 2], and OCTA [Table 3] imaging modalities. While these features are not specific to CVD and can occur in other microvascular conditions such as type 2 diabetes mellitus, human immunodeficiency virus, and leukemia, we highlight their established associations with CVD. We delineate these biomarkers into metrics, markers that can be measured in all eyes, and abnormalities, unusual phenomena or patterns whose presence may signify underlying health concerns. Many of the biomarkers highlighted in this section arise from vascular insults manifesting in the retina. Like microvascular damage in other organs, the retina is vulnerable to endothelial dysfunction resulting from impaired perfusion and capillary rarefaction [31,32,33], shear stress alterations from systemic hypertension [31,33], neurovascular decoupling due to inadequate oxygen and nutrient delivery [34], and oxidative stress and inflammation secondary to ischemia [31,33]. These systemic vascular insults, stemming from disease external to the eye, culminate in the diverse forms of retinal dysfunction captured by the imaging biomarkers discussed.

### 4.1. Color Fundus Photography Metrics

#### 4.1.1. Arteriovenous Nicking

**Figure 5:**
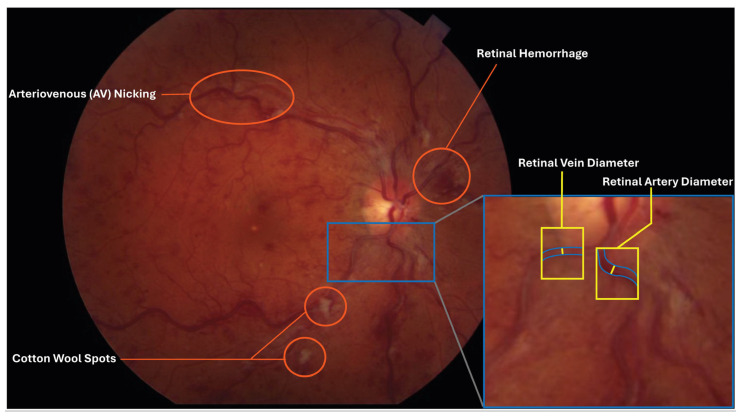
A color fundus photography (CFP) image of a patient presenting with several CFP biomarkers associated with cardiovascular disease highlighted with red circles, including arteriovenous (AV) nicking, cotton wool spots (CWSs), and a retinal hemorrhage. A zoomed-in segment of the image illustrates how retinal vein diameter and retinal artery diameter are measured.

Arteriovenous (AV) nicking, a finding observed on CFP, is when an arteriole is observed crossing a venule, which results in indentation and compression of the venule [Figure 5].

#### 4.1.2. Retinal Vessel Caliber, Arteriovenous Ratio, Central Retinal Arteriole Equivalent, and Central Retinal Venular Equivalent

Retinal vessel caliber, a measure of the diameter of blood vessels in the eye, is a critical CFP measurement. From this, a variety of measures can be calculated, including arteriovenous (A/V) ratio, central retinal arteriolar equivalent (CRAE), and central retinal venular equivalent (CRVE). The A/V ratio is a ratio between the relative caliber of retinal arteries in comparison to retinal veins, with retinal arteries typically narrower than veins. The CRAE reflects the average diameter of the arterioles present in the retina, while the CRVE reflects the average diameter of the venules present in the retina.

#### 4.1.3. Retinal Arteriolar and Venule Diameter

Static retinal vessel analysis (SRVA) allows for the measurement of retinal arteriolar diameter through the use of single digital images of the fundus. Retinal arteriolar and venule diameter are systematically measured from this image and reflect the state of the vessel at the time of image capture, indicating whether it is wide or narrow [35]. However, if widening or narrowing of these vessels is observed, SRVA cannot distinguish whether the root cause is physiological or pathophysiological [35].

Vessel diameters are calculated by analyzing the brightness profile of a segment of the vessel. In this method, markers are placed on either side of the vessel edges [Figure 5]. The diameter is calculated perpendicular to the direction of flow between the vessel edges [Figure 5]. The diameter itself is not the entire lumen between the vessels but rather where the column of flowing blood cells is positioned. This is done throughout the vessel so different diameters can be correlated to different positions within the retina.

### 4.2. Color Fundus Photography Abnormalities

#### 4.2.1. Retinal Hemorrhage

Retinal hemorrhages can range from dot and blot hemorrhage to massive sub-hyaloid hemorrhages [Figure 5] and can be characterized by their location, size, and distribution to determine underlying causes, including vascular disease, hematologic disorders, dyscrasias, infections, trauma, and hypoxia [36].

#### 4.2.2. Cotton Wool Spots

CWSs are discrete white lesions that lie within the retinal nerve fiber layer. These lesions are thought to represent nerve fiber layer infarct caused by pre-capillary arteriolar occlusion [Figure 5]. On their own, CWSs are asymptomatic and usually resolve within 6–12 weeks, although they may persist in diabetic patients [37]. They can often leave behind areas of retinal thinning, which may cause persistent blind spots identified using visual field tests [38]. However, while often asymptomatic, CWSs generally coincide with other ocular and systemic vascular disorders.

### 4.3. Optical Coherence Tomography Metrics

#### 4.3.1. Retinal Nerve Fiber Layer Thickness

After the advent of the original spectral domain OCT (SD-OCT), retinal layer thicknesses were often assessed, given its ability to analyze backscattered light from the retinal tissue [39,40]. The retinal nerve fiber layer (RNFL) has been a layer of interest, as considerable evidence indicates that RNFL thinning precedes vision loss in glaucoma [41,42,43,44,45,46,47,48,49], an ocular disease caused by increased intra-ocular pressures leading to optic nerve damage. After obtaining a thorough understanding of normal values for RNFL thickness, it has become a mainstay as a biomarker of retinal damage.

#### 4.3.2. Subfoveal Choroidal Thickness

Although the traditional SD-OCT scans were able to clearly identify retinal layers, their resolution of anatomy posterior to the RPE, such as the choroidal vasculature, was limited. With the advent of newer OCT technology, such as enhanced depth imaging (EDI) SD-OCT [50] and swept-source OCT (SS-OCT), visualization of the choroid, a vasculature bed between the RPE and sclera, is now possible, and its thickness can be measured [51,52,53,54]. The choroid is an especially important retinal vascular bed, as it receives the vast majority of ophthalmic artery blood flow, nourishes and oxygenates the outer retinal layers, and serves as a cooling mechanism for the fovea, the primary retinal location of focused light [55,56,57]. Of note, studies have identified choroidal changes in ocular pathology, including AMD and glaucoma [57,58,59,60,61,62,63,64,65,66,67,68].

### 4.4. Optical Coherence Tomography Abnormalities

#### 4.4.1. Paracentral Acute Middle Maculopathy Lesions

**Figure 6:**
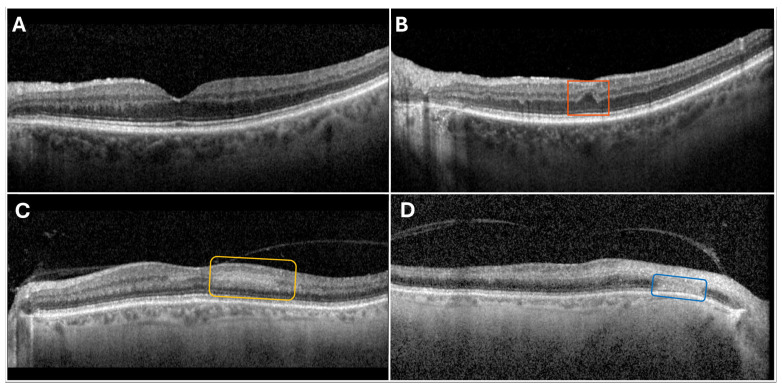
Representative optical coherence tomography (OCT) B-scans illustrating the appearance of (**A**) a normal eye, (**B**) a retinal ischemic perivascular lesion (RIPL; outlined in orange), (**C**) paracentral acute middle maculopathy (PAMM; outlined in yellow), and (**D**) an acute macular neuroretinopathy lesion (AMN; outlined in blue; courtesy of Ria Desai, MD).

PAMM lesions are an OCT finding seen in patients with acute retinal ischemia. Even though they can present independently, PAMM lesions often arise due to retinal damage due to some form of systemic vascular disease [69,70,71]. PAMM lesions can be defined as diffuse hyperreflective bands at the level of the INL [69,70,71] [Figure 6].

#### 4.4.2. Retinal Ischemic Perivascular Lesions

RIPLs are small retinal lesions secondary to chronic focal mid-retinal hypoperfusion [72,73]. They are identified when analyzing the retinal layers on OCT imaging characterized by outer plexiform layer (OPL) displacement, outer nuclear layer (ONL) expansion, and INL thinning [Figure 6]. They were initially discovered in hypertensive patients, but their correlation with other cardiovascular diseases that may cause retinal ischemic disease has been further explored [73].

#### 4.4.3. Subretinal Drusenoid Deposits

**Figure 7:**
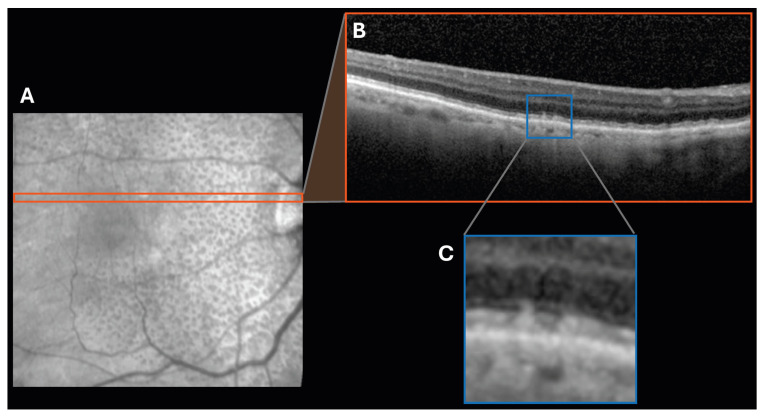
Subretinal drusenoid deposits (SDDs), also called reticular pseudodrusen (RPD), identified on an (**A**) optical coherence tomography (OCT) en face image with (**B**) a corresponding B-scan at this location. (**C**) A zoomed-in segment of the B-scan highlights the SDDs.

Age-related macular degeneration (AMD) is one of the leading causes of blindness amongst older adults [74]. There are two clinical presentations of AMD: non-exudative (dry) AMD and exudative (wet) AMD. Dry AMD, commonly presenting with drusen, yellow pockets of lipids and protein located between the retinal pigment epithelium (RPE) and Bruch’s membrane (BM), can also present with subretinal drusenoid deposits (SDDs), protein deposits located above the RPE but below the retina [Figure 7]. SDDs are also known as reticular pseudodrusen, although we will refer to them solely as SDDs in this review [75,76].

SDDs are commonly seen in AMD but can also exist independently in other conditions, including IgA nephropathy [77], vitamin A deficiency [78], and macular atrophy [79]. Given their association with various systemic and cardiovascular diseases manifesting in the eye, SDDs are emerging as a crucial biomarker of both retinal and systemic health.

### 4.5. Optical Coherence Tomography Angiography Metrics

#### 4.5.1. Foveal Avascular Zone

**Figure 8:**
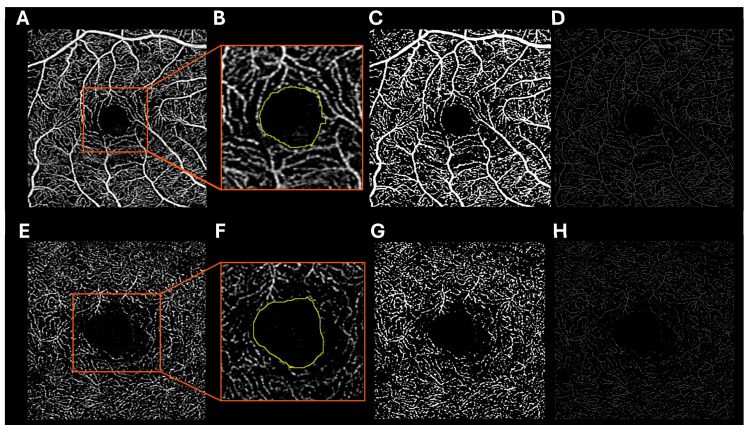
Swept-source optical coherence tomography angiography (SS-OCTA) representative images of the (**A**) superficial capillary plexus (SCP) and (**E**) deep capillary plexus (DCP) with additional images demonstrating (**B**,**F**) measurement of the foveal avascular zone (FAZ; yellow outline), (**C**,**G**) binarization of the vessels for calculation of vessel area density (VAD), and (**D**,**H**) skeletonization of the vessels for calculation of vessel length density (VLD).

The foveal avascular zone (FAZ) is the central region of the retina that is devoid of blood vessels and overlying inner retinal tissue [Figure 8]. Common FAZ measurements include the area of the avascular region (FAZ area), the length of the capillary ring surrounding the region (FAZ perimeter), and the roundness of the region (FAZ circularity, calculated as 4π × (FAZ area)/(FAZ perimeter)^2^).

#### 4.5.2. Vessel Area Density

The vessel area density (VAD) is the ratio of the retinal area covered by blood vessels to the total retinal area, often excluding the foveal avascular zone, in a particular en face image [Figure 8]. The VAD can be measured and differs at each capillary plexus due to anatomical differences, such as tissue thickness and vessel branching patterns, and differing functional demands of the tissues they provide.

#### 4.5.3. Vessel Length Density

The vessel length density (VLD) is the ratio of the total length of 1-pixel-wide skeletonized blood vessels to the total retinal area, generally excluding the foveal avascular zone [Figure 8]. The VLD can be measured and differs at each capillary plexus. By measuring specifically the length of the retinal vessels, VLD represents the degree of branching within the retinal microvasculature.

These retinal imaging biomarkers and metrics, initially established to explore local retinal diseases, have been powerful tools to explore the impact of systemic vascular disease on the retina. Furthermore, it is now apparent that the retina provides a unique and accessible window into the systemic microvasculature. Below we discuss the relationship of these metrics with various forms of CVD and general CVD risk scores.

## 5. Association of Retinal Biomarkers with Cardiovascular Diseases

### 5.1. Hypertension

Uncontrolled hypertension and its associated organ damage are considered prominent risk factors for cardiovascular disease. Chronic hypertension is associated with increased vascular resistance, and as a result has been found to correlate with narrower retinal arteriolar calibers, wider venular diameter, and smaller arteriole-to-venule diameter ratio. Those with the narrowest arterioles or the widest venules also had an increased risk of developing hypertension [80]. Wider retinal venules and narrower retinal arterioles were also associated with increased risk for developing cardiovascular disease, correlating specifically with diabetes presence, being on antihypertensive medications, higher systolic blood pressure, higher total cholesterol, and lower HDL cholesterol [81]. A meta-analysis further solidified the link between retinal arteriolar diameter and hypertension, demonstrating a significant inverse relationship between arteriolar caliber and blood pressure [82]. The presence of AV nicking was increased in hypertensive individuals despite the use of antihypertensive medications [83].

Although many vascular sequelae due to hypertension exist, not much evidence in the literature points to structural retinal changes that can be appreciated using OCT imaging. However, a recent finding describes increased prevalence of RIPLs in chronic hypertensive patients, seen in patients with mild HTN as well, indicating this biomarker may be a useful indicator of subclinical CVD [84].

Following well-established correlations between poorly controlled hypertension and decreases in retinal perfusion, several studies have explored the retinal manifestations of hypertension using OCTA images. Many studies have associated hypertension with decreased VAD and VLD in the DCP and SCP layers of the retina, particularly in the macula [34,85,86,87,88,89,90,91,92]. One of these studies further divided their HTN patients into those with HTN retinopathy and those without and found a consistent decrease in vessel density regardless of the presence of HTN [34]. However, there are some negative studies regarding either the decrease in SCP VLD [85,86,87,91] or DCP VLD [92] of hypertensive patients. Similar trends have been found regarding FAZ area, with multiple studies finding associated decreases in both SCP and DCP FAZ area [85,88,89,92].

Studies have further explored this relationship between the retina microvasculature and hypertension by exploring the effects of HTN duration, concurring CV risk factors, and blood pressure control. While the effect of HTN duration has yielded contradictory results [89,92], HTN patients with more CV risk factors have demonstrated decreased VLD in both the SCP and DCP macular regions [90], and HTN patients with higher blood pressure presented with significantly decreased DCP VAD compared to HTN patients with well-controlled blood pressure [86,87]. Taken together, these studies indicate the potential for using OCTA parameters to monitor the severity and progression of HTN.

### 5.2. Coronary Artery Disease and Atherosclerotic Disease

Decreases in the retinal microvasculature density have been correlated with the presence of coronary artery disease (CAD) and traditional CAD metrics. Rates of coronary heart disease events, ischemic strokes, heart failure, and death were all found to be higher in individuals with wider retinal venules and narrow retinal arterioles [81]. Atherosclerotic disease of retinal and cardiac arteries provides a pathophysiologic link between these findings. The internal carotid artery can often be occluded due to atherosclerosis and often results in ocular ischemic syndrome, presenting with visual loss, orbital pain, and changes in the visual field. Findings specific to the retina include narrowed retinal arteries, dilated retinal veins, retinal hemorrhages, microaneurysms, cotton wool spots, and neovascularization, all of which are visible on CFP [93].

When comparing the OCT-derived structural metrics for a group of patients after myocardial infarctions in comparison to healthy controls, RIPLs were increased in patients with myocardial infarctions [94]. Studies indicate there is a strong correlation between CAD/MI and SDD presence, indicating there is a pathophysiologic link between both conditions [95,96]. Wang et al. performed a statistical correction for age and sex and refined inclusion criteria to minimize confounding from uncontrolled HTN and T2DM, thus strengthening the association between retinal structural changes identified on OCT, such as SDDs, and CAD. In contrast, Cymerman et al. discussed age, gender differences, smoking status, and hypertension in the context of CAD progression but did not statistically adjust for these risk factors. This may suggest that SDDs are an indicator of wider, systemic disease in the AMD population, but given the differences in study organization, more extensive research needs to be conducted to solidify this finding. This may suggest that SDDs are an indicator of wider, systemic disease in the AMD population. Patients with CAD also exhibit decreased choroidal thickness in comparison to healthy controls [97]. These studies indicate atherosclerosis has retinal manifestations, a key finding given the emerging relationship between the retinal and cardiac vascular beds, as studies have associated myocardial perfusion metrics identified on PET/CT imaging with retinal vessel metrics derived from macular OCTA imaging [98,99].

When assessing OCTA-derived vascular metrics, decreases in SCP and DCP VAD have been correlated to various manifestations of CAD, including chronic three-vessel disease and myocardial infarction [100,101]. The extent of left main coronary artery (LMCA), left circumflex coronary (LCX) artery, and right coronary artery (RCA) stenosis is correlated to decreased RPCP, SCP, and CC VAD [96]. These associated decreases in retinal vessel density also correlated to worsening LVEF [102,103].

### 5.3. Valvular Heart Disease

Valvular heart disease often causes downstream hypoperfusion and ischemic sequelae given the chronic inefficiencies in systemic vascular perfusion. In patients with AMD, OCT-centric studies have shown that patients with nondescript valvular heart disease have increased SDDs in comparison to clinical controls. SDD formation in this population is hypothesized to be secondary to retinal hypoperfusion in valvular heart disease [104].

More specifically, early diagnosis and treatment of aortic valve regurgitation (AR) can help prevent hypertrophy of the cardiac muscle and further complications. OCTA imaging may allow for early identification of possible AR, as significant and specific decreases in retinal vessel density have been associated with AR diagnosis. In AR patients, SCP, DCP, and CCP VLD all decreased [105]. While more research is needed to further elucidate this relationship, this study points to the potential for OCT and OCTA imaging to aid in early AR detection.

### 5.4. Cerebral Infarctions

Although cerebral infarctions are described as acute adverse cardiac events, not chronically managed diseases, significant exploration has been done to unveil whether retinal imaging can better assess stroke incidence. In color fundus studies, patients without a history of stroke or coronary heart disease with retinal hemorrhages during a 20-year follow-up had an increase in stroke incidence. As previously mentioned, RVOs, identified on CFP or OCT imaging, are heavily correlated with future stroke incidence [29]. Structural retinal biomarkers such as RIPLs are significantly increased in patients with RVOs [106] and have been associated with single subcortical stroke and cerebral small vessel disease [107]. In conjunction with subcortical stroke, in a cohort of atrial fibrillation patients, individuals with increased stroke rates had higher RIPL quantifications in comparison to unaffected controls [108]. This evidence further strengthens the relationship between retinal ischemia and cerebral ischemia.

Stroke occurrence and coinciding severity metrics have also been correlated to OCTA parameters. After adjusting for age and sex, stroke patients have consistently demonstrated significantly reduced macular SCP VAD, although one study found this reduction was insignificant when accounting for cardiovascular risk factors [109,110]. Additionally, DCP and RPCP VAD [110] as well as DCP FAZ circularity [111] show a significant reduction in ischemic stroke patients, suggesting stroke occurrence may simply decrease the overall retinal microvasculature perfusion. Notably, SCP VAD has also been negatively correlated to the presence of mild cognitive impairment resulting from cerebral infarction [112] and the level of MRI-measured white matter hyperintensities [113]. These studies suggest a potential role for OCTA in monitoring the severity of cerebral infarction symptoms.

### 5.5. Atrial Fibrillation

Atrial fibrillation has the potential to cause acute, focal retinal ischemia due to clot formation, similar to acute ischemic strokes and other peripheral vascular sequelae. As an indicator of retinal ischemia, RIPLs have been shown to be increased in atrial fibrillation, a common source of embolic ischemia [114].

### 5.6. Carotid Artery Disease

The buildup of carotid plaque and the progression of CAS have shown worsening in retinal microvascular dysfunction. Studies have indicated that CAS severity and worsening clinical status are correlated with the presence of RIPLs [115,116]. CAS has been correlated with ipsilateral SDDs and choroidal thinning, suggesting reduced blood flow from ICA stenosis leads to SDD formation [117]. As a result, RIPLs and SDDs could be valuable, independent biomarkers for detecting downstream vascular insults secondary to CAS.

PAMM lesions are linked to retinal vascular disorders such as CRAO, CRVO, BRAO, and hypertensive disease, all of which are significantly correlated with CAS [118,119,120,121]. Although isolated cases of PAMM lesions have been reported in the literature [122], isolated PAMM lesions without additional ocular pathology may not necessarily suggest an elevated cardiovascular risk.

The presence of CAS has also been correlated to a decrease in RPCP and SCP VAD when compared to healthy controls [123]. This decrease in SCP VAD may be greater in the eye ipsilateral to the stenosis compared to the contralateral eye [124]. Similarly, the same study found a greater FAZ area in ipsilateral eyes [124]. These findings specific to the ipsilateral eye demonstrate the importance of monitoring differences in retinal microvascular metrics between eyes. In addition, there is a negative correlation between carotid plaque width and SCP and DCP VAD [125]. In contrast, carotid artery stenting and angioplasty have demonstrated improvement in DCP VLD following the procedure [126]. These studies show an ability to monitor the severity and improvement of CAS after diagnosis and treatment using retinal imaging.

### 5.7. Heart Failure

Heart failure (HF), a complex syndrome characterized by structural or functional impairments of ventricular filling or ejection, is caused by cardiac ischemia, infection, anatomical stress, or pharmacologic side effects. In the context of lowered ejection fraction, there is a risk of end-organ damage due to decreased perfusion. Multimodal retinal imaging offers a noninvasive view into microvascular changes secondary to the macrovascular dysfunction in HF [127,128,129].

The Atherosclerosis Risk in Communities Study found that a wider CRVE and a narrower CRAE, after adjusting for age, gender, and race, were associated with HF development as well as worse systolic and diastolic function [130]. Additionally, wider CRVE is associated with 12-month HF rehospitalization and mortality [131]. The Australian Heart Eye Study reported significant retinal arteriolar changes in HF; however, they indicated a wider retinal arteriolar caliber was associated with higher odds of developing HF, with further increased odds in diabetic patients [132]. Given that HF status was self-reported, misclassification in disease status could have occurred, erroneously skewing results [132]. Of note, patients with diabetic retinopathy (DR) had a higher incidence of congestive heart failure (CHF) compared to those without retinopathy [133]. After controlling for age, sex, race, preexisting coronary heart disease, mean arterial blood pressure, diabetes, glucose, cholesterol, smoking, and BMI, DR presence remained associated with congestive heart failure at a 2-fold higher risk [133]. DR can be diagnosed with CFP, and notable findings include AV nicking, neovascularization, microaneurysms, retinal hemorrhage, cotton wool spots, and hard and soft exudates [127]. Therefore, timely DR identification may prompt physicians to pursue cardiac workups given increased HF odds.

Retinal vessel analysis (RVA), an alternative retinal imaging analysis that captures flicker-induced dilatation of retinal arterioles using a fundus camera, can identify retinal regions of impaired arteriolar dilation, indicative of retinal microvasculature and endothelial dysfunction [134]. Notably, RVA analysis identified impaired retinal arteriole dilation in congestive HF patients in comparison to controls [135,136]. These findings demonstrate CFP’s great potential in characterizing retinal microvasculature changes that occur during HF.

When assessing OCT metrics in HF, studies have explored retinal alterations in chronic disease profiles such as HF with preserved ejection fraction (HFpEF) or HF with reduced ejection fraction (HFrEF) in addition to retinal microvasculature changes in acute decompensations (ADHF) as well. In HFpEF, patients had decreased macular ganglion cell-inner plexiform layer thickness and total retinal volume [137]. In HFrEF, patients had a lower subfoveal choroidal thickness (SFCT) compared to controls [138,139,140]. Additionally, after stratifying for HFrEF patients with an ejection fraction (EF) <30%, a significant decrease in the RNFL thickness was also appreciated [140]. In ADHF patients during the acute phase, a decrease in SFCT and supertemporal retinal vein and artery diameter was seen in comparison to controls [141]. Only the SFCT decrease remained after ADHF resolution. Of note, no changes were seen in the central macular thickness during or after an ADHF event. Lastly, no significant OCT changes, specifically in the ganglion cell complex, were identified in chronic HF patients due to dilated cardiomyopathy in the pediatric population [142]. Interestingly, studies have shown a 36% increased odds of HF, more so ischemic HF, after an RVO event, similar to cerebral infarctions [143].

While OCTA studies in HF patients are limited, current research has already demonstrated OCTA’s potential for monitoring HF and reduced ejection fraction progression. Multiple studies have found consistent decreases in SCP VAD and VLD in HF patients compared to controls [138,141,142]. One study further delineated a significant reduction in SCP VAD between HF patients categorized as group 2 according to New York Heart Association criteria compared to group 3 HF patients [139], illustrating OCTA’s utility in monitoring HF risk or progression. This same study also found a significant reduction in DCP VAD between group 2 and group 3 patients as well as between controls and group 3 patients [139]. OCTA imaging has also been studied to assess vascular metric changes to capture vascular recovery in patients hospitalized with ADHF. When comparing ADHF patients imaged within 24 h of admission and healthy controls, the study reported a significant reduction in macular SCP VLD. Notably, when the same ADHF patients were imaged at discharge after IV diuretic treatment, patients had a significant increase in macular SCP VLD [141]. Given these findings, multimodal retinal imaging may reflect current systemic vascular health in HF patients but can also provide information on the status of HF patients as they undergo treatment, start medications, or acutely decompensate.

### 5.8. CVD Risk Scores and Generalized Risk Factors

Much research has explored the connection between retinal imaging and nondescript CVD risk or CVD risk scores to further define retinal imaging’s relationship to CVD risk. A color fundus finding that has received particular attention as a potential biomarker for general CVD risk is cotton wool spots. CWSs conferred an increase in stroke risk following the initial presentation [144]. Even when controlled for other cardiovascular risk factors, CWSs were associated with an increased risk of congestive heart failure and sequelae [145,146].

CFP has also shown promise in cardiovascular risk prediction as a supplement to the existing pooled cohort equations. Specifically, after adjustment for the pooled cohort equation-predicted risk score, wider retinal venules and narrower retinal arterioles were correlated to an increased risk of death and stroke in both sexes and an increased risk of coronary heart disease in women [81]. Thus, CFP-derived retinal biomarkers may increase the predictive ability of pooled cohort equations on their own.

When analyzing the relationship between OCT-derived structural metrics and CVD risk, numerous biomarkers have identified relationships with CVD risk. RIPLs have been shown to be elevated in patients with nondescript CVD and are also increased in patients with higher ASCVD risk scores in comparison to low ASCVD risk scores [73,147,148]. Additionally, decreases in RNFL thickness have been associated with increased risk of generalized cardiovascular events, including coronary heart disease, heart failure, stroke, and general CVD-related mortality [149].

Subfoveal choroidal thickness has also been found to decrease when comparing high- and low-risk individuals, as identified by CAC scores [150]. A reduction in foveal choroidal thickness is also observed in patients with coronary artery disease and acute coronary syndrome [97] along with other CVD risk factors, including systolic blood pressure, eGFR, dyslipidemia, and LV-End Diastolic Pressure [151]. However, the utility of subfoveal choroidal thickness as a predictive metric in clinical practice is limited, as variables such as age and axial length significantly influence subfoveal choroidal thickness measurements [151,152,153].

As previously mentioned, patients with AMD and SDDs are highly correlated with risk factors and diagnoses indicating systemic cardiovascular dysfunction, including hypertension, smoking, CAD, and angina [154]. Studies have found a significant increase in cardiovascular risk factors and high-risk vascular disease (MI, CHF, and valve defects) correlating to an increase in SDDs and significant choroidal thinning [155,156].

Analysis of the relationship between various CVD risk scores and OCTA microvasculature parameters has yielded mixed results. One study identified a correlation between a high AHA risk score and decreased overall retinal vessel density parameters, particularly SCP VAD, but also found a negative relationship between VAD metrics and GRACE (Global Registry of Acute Coronary Events) scores [102]. Another study found correlations between various 10-year risk scores, including ASCVD risk, GRACE score, Reduction of Atherothrombosis for Continued Health (REACH) score, and Thrombolysis in Myocardial Infarction (TIMI) risk score, and choriocapillaris flow void parameters, including decreased count, increased average size, and increased signal void area [157]. However, this study found no correlation between SCP and DCP VAD and these risk scores [157]. More research on retinal density parameters in CVD risk scores is needed to elucidate if OCTA may help in the early identification of high cardiovascular risk patients.

The thorough and continuously expanding exploration into these biomarkers and vascular metrics is valuable in developing a pathophysiologic foundation connecting the retina and the systemic vasculature. Transitioning these findings from research to the clinic is an area of interest, but progress has been limited given manual biomarker quantification and limited actionable clinical conclusions from these biomarkers. However, artificial intelligence provides an avenue for advancing clinical implementation on multiple fronts.

## 6. Artificial Intelligence in Retinal Imaging for CVD Risk Prediction

The recent development of deep learning (DL) algorithms, more specifically convolutional neural networks (CNNs), in medical image analysis is quickly proving to be a promising method for the detection of retinal biomarkers [158]. These algorithms enable numerous retinal vascular parameters to be automatically detected and applied towards the calculation of objective measurements. The ability to attain these objective parameters with great efficiency is a crucial development for the future of cardiac-oculomics research.

The development of DL programs specific to retinal image analysis has been aided by the availability of large public datasets with accurate and consistent labeling and good image quality. A 2021 review found 94 open-access retinal image datasets, yielding a total of 507,724 images from at least 122,364 individuals [159]. Of the 94 identified open-access datasets, 54 were CFP datasets, 18 were OCT/OCTA datasets, 7 were external eye image datasets, and the other 15 datasets consisted of various imaging modalities, including fluorescein angiography and confocal microscopy, among others [159]. These datasets provide diverse patient population imaging for adequate training and validation for DL models.

### Role of AI in Analyzing Retinal Images

The first DL programs used in retinal image analysis focused on grading image quality, identifying common diagnoses, and segmenting retinal biomarkers [160,161]. However, the key to the practicality of AI in the discovery of retinal manifestations of cardiovascular disease lies in the segmentation of retinal biomarkers. Multiple algorithms have already been developed for the segmentation and quantification of retinal parameters, including retinal vessel caliber measurement and arterial and venule classification from color fundus imaging, layer thickness quantification from OCT imaging, as well as vascular density and FAZ measurements from OCTA imaging [162,163,164,165,166,167,168,169]. Further development of automated vessel segmentation algorithms will pave the way for new discoveries of retinal biomarkers for cardiovascular disease. Nevertheless, many researchers have already been utilizing these AI models across several retinal image CVD risk prediction studies.

Using a DL algorithm trained on data from 284,335 patients and validated with two independent datasets of 12,026 and 999 patients, Poplin et al. found that they could predict several CVD risk factors from color fundus images alone, notably age (mean absolute error within 3.26 years), gender (AUC = 0.97), smoking status (AUC = 0.71), systolic blood pressure (mean absolute error within 11.23 mmHg), and major adverse cardiac events (AUC = 0.70) [170]. Meanwhile, Zhang et al. achieved high accuracy for the prediction of chronic conditions associated with CVD risk using a DL model trained on only 1222 color fundus images. Specifically, their model predicted hypertension, hyperglycemia, and dyslipidemia with accuracies of 68.8%, 78.7%, and 66.7%, respectively [171]. Similarly, Chang et al. trained a DL model to predict atherosclerosis from fundus images with an accuracy of 58.3%. In their retrospective cohort, they also found a significant association between the DL-predicted presence of atherosclerosis and CVD mortality [172].

Groups have also found success in predicting CAC scores from retinal fundus images using DL models. One group concluded that their DL-and-fundus-image-derived CAC scores were comparable to CT-derived CAC scores in predicting cardiovascular events. Rim et al. trained a DL model on 216,152 fundus images from the United Kingdom, South Korea, and Singapore to predict the probability of CAC presence. Ultimately, the researchers concluded that these retinal image-based DL CAC scores (RetiCAC) were comparable in 10-year CVD risk prediction to the traditional CT-derived CAC scores. The same research group further validated this model by assessing its ability to predict CVD events in the general population. Within their cohort of 48,260 patients, the high RetiCAC score group was associated with a 13.1% 10-year CVD risk [173]. Conventionally, a 10% 10-year CVD risk indicates clinical intervention. Therefore, this association confirms RetiCAC as a reliable biomarker for identifying individuals with a high 10-year CVD risk where early intervention may be beneficial. On a larger scale, Zhou et al. developed a foundational retinal AI model (RET-Found) by training the model on 904,170 CFP and 736,442 OCT unlabeled images [174]. This large training dataset allows RETFound to be applied to various downstream tasks, including systemic disease prediction. Zhou et al. tested RETFound on four systemic disease prediction tasks. Through this, RETFound achieved an AUC of 0.737 for the prediction of myocardial infarction, an AUC of 0.794 for the prediction of heart failure, an AUC of 0.754 for the prediction of stroke, and an AUC of 0.669 for the prediction of Parkinson’s disease, all from CFP [174]. It performed similarly on prediction of these diseases from OCT images [174].

In discussing these burgeoning retinal image AI models, we seek to highlight the future potential of retinal AI applications. At the current stage, the models have not reached a level of accuracy warranting clinical use. Further research is needed to rigorously test inter-device reproducibility, standardize imaging and segmentation protocols, and ensure diverse participant representation in datasets. Thus, while these studies have demonstrated considerable promise for AI implementation in cardiac-oculomics, more research and validation of these DL algorithms is needed to confirm the reliability of these models.

## 7. Clinical Implications of Retinal Imaging in Cardiovascular Disease

### 7.1. Integration into Routine CVD Risk Assessment

#### 7.1.1. Feasibility of Retinal Imaging in Clinical Practice

Given the ability of retinal imaging modalities, including CFP, OCT, and OCTA imaging, to identify ocular manifestations of CVD, there are numerous opportunities for integration into the clinical environment outside of ophthalmology. With evolving AI and imaging technology, these forms of imaging can be acquired non-mydriatically or with smartphones [175,176,177,178,179,180,181,182], allowing access to retinal information with limited ocular intervention regardless of past medical history. With the advent and further optimization of various AI models and automatic post-processing technology, various vascular metrics and clinical conclusions can be obtained autonomously without ophthalmology personnel actively needed for post-processing and analysis. Additionally, as AI techniques continue to evolve and a singular pathway from imaging to cardiovascular risk assessment is complete, retinal imaging could be a clinical mainstay in cardiology and primary care clinics for its inexpensive, noninvasive clinical utility.

The primary drawbacks of implementing ocular imaging in the cardiology clinical environment are the technology, personnel, and cost associated with retinal image acquisition. To offset such a cost, ocular imaging would need to have a strong predictive value and serve a unique and valuable need for cardiologists. Given the evidence above and the quick innovation in cardiac-oculomics, a well-developed clinically applicable pipeline and further evidence comparing retinal imaging to the status quo of CVD risk prediction are needed to justify the expense. Further studies must be undertaken to better understand the clinical utility of multimodal retinal imaging and build a workflow or pipeline to use it in clinical practice. Regardless, this technology has the powerful and unique ability to provide a viewpoint into a patient’s vascular health, which has not yet been as easily accessible.

#### 7.1.2. Accessibility in Lower Resource Settings

The clearest advantage of retinal imaging would be harnessed in lower-resource settings, such as in patients with limited access to consistent clinical care, as retinal images can now be accessed through smartphone cameras [181,183,184,185]. In addition, retinal imaging provides a wealth of quality information with regard to CVD risk prediction, providing a viewpoint into the pathophysiologic progress of ocular and cardiovascular diseases [98,99,183]. It could serve as a feasible complement to CVD risk prediction and systemic vascular health. In populations where follow-up may be difficult, cost-associated limitations hinder further diagnostics, and technologically intensive diagnostic and risk prediction metrics are not available, ocular imaging would offer the most value for patients and physicians. Although risk prediction metrics exist to triage and guide cardiovascular next steps, such as hypertension, hemoglobin A1c, and other ASCVD metrics, retinal imaging is uniquely accessible and noninvasive while detecting early and minute microvascular changes, offering a glimpse into the health of the vascular system before overt clinical symptoms of cardiovascular disease manifest. Furthermore, there is evidence correlating retinal imaging with complex imaging such as PET/CT stress tests [98,99], and coronary calcium scores [150,183], which provides patients with rich, predictive risk metrics that can all be performed in-office, a valuable component of integrating retinal imaging in the cardiology environment. This synergistic approach may ultimately lead to more precise cardiovascular risk management, improving long-term cardiovascular health outcomes in patients who previously may not have had access to this information.

### 7.2. Personalized Medicine and Patient Management

#### Stratification of CVD Risk and Assessing Cardiovascular Status to Monitor Disease Evolution

Personalized medicine is a generalized term to describe adapting healthcare towards the acute and unique background and characteristics of each patient. When offering diagnostics, treatments, or procedural interventions, physicians already practice personalized medicine, as physician recommendations are rooted in a patient’s medical history, imaging, or other clinical data. Risk prediction metrics encompass the eventual goal of personalized medicine, a standardized and quantitative tool to offer specific, actionable advice to patients based on their clinical data. Currently, risk prediction metrics provide valuable data for patients, but they are generally static and not specific enough to a patient’s acute vascular dynamics without the use of imaging. Imaging the retinal vasculature is the only real-time opportunity for clinicians to analyze acute changes to a patient’s microvasculature in response to disease progression or treatment initiation, providing more context to the systemic implications of a patient’s current clinical status [186,187]. Retinal imaging, in conjunction with larger systemic variables, such as physical exam findings and lab values, has the potential to be used to acutely assess cardiovascular status and modulate treatments for patients with CVD, as seen previously in retinal microvasculature alterations due to antihypertensive medications [188,189,190]. Additional research and model creation need to be performed to realize such a goal, but retinal imaging has the unmatched potential to provide a novel, personalized point of view into the development and treatment of cardiovascular disease.

## 8. Challenges and Limitations

### 8.1. Technical and Methodological Challenges

#### 8.1.1. Variability in Imaging Techniques

Variability in retinal imaging techniques poses a significant challenge in ensuring consistent and reliable results across different machines, operators, and healthcare settings [Table 4]. This variability can stem from differences in hardware specifications, software algorithms, and operator expertise, leading to discrepancies in image quality and interpretation [191,192,193]. However, as cardiac-oculomics continues to gain significant ground in the field of precision medicine, numerous techniques have already been implemented and will continue to be developed to counteract these obstacles. Standardization of image acquisition protocols, specifying parameters such as image resolution, scan patterns, operator training, and patient preparation, can significantly enhance the reproducibility of retinal imaging studies. Continuing to increase awareness of the field of cardiac-oculomics is critical towards fostering interdisciplinary collaboration to drive the development of robust validation studies and clinical practice guidelines for use in patient care.

#### 8.1.2. Interpretation and Training

Moreover, the implementation of quality control measures, such as regular calibration of imaging devices and cross-validation of images, can help maintain consistency. Advances in AI and machine learning also offer promising solutions for reducing variability by automating image analysis and minimizing operator bias. AI and machine learning have already obtained a strong foothold in various other fields of medicine, allowing for the seamless application of lessons learned from these domains to accelerate progress in cardiac-oculomics. Continued investment in this tool in the form of diverse and specific AI training sets, comprehensive quality control measures, and the creation of a relatable user interface are all achievable goals in which significant progress is already underway and will continue to be made. Ensuring consistency across different machines and settings not only improves the reliability of retinal imaging but also enhances its utility as a diagnostic and monitoring tool in clinical practice.

### 8.2. AI-Related Challenges and Ethical Limitations

While the rapid development of DL models and their application to retinal image CVD risk assessment shows great promise for future healthcare practice, this burgeoning field brings with it important ethical considerations prior to implementation [Table 4]. Evans et al. identified three main ethical concerns for the expansion of AI: model transparency, attribution of responsibility, and scalability of use cases and infrastructure [194].

AI model transparency becomes pivotal when models produce a known false answer or malfunction. While some models are interpretable, many DL algorithms are too complex for human comprehension. These “black box” algorithms require post-hoc explanation, or else these erroneous outputs could negatively impact patient care or research output.

As AI models become prominent in clinical care, determining which arm of AI-assisted medical care, the provider or software company, is responsible for patient harm resulting from inaccurate AI outputs is paramount. Often the companies designing the AI models assume responsibility, as they define the specific criteria in which AI software may be used. However, whether the providers should be held responsible for determining the appropriate use of these models is unclear.

Finally, there are also multiple issues related to the scalability of AI models [Table 4]. Scalability can be limited if training datasets do not fully encompass the population that the model may be applied to. For example, poorly diversified AI models may disproportionately misdiagnose certain conditions among disadvantaged groups who may not have been properly represented in the training datasets. This bias in medical technology has already been seen in the introduction of pulse oximeters [195] and radiograph datasets [196]; and, given the current limitations of large-scale dataset availability for training AI models, the likelihood that there is bias in AI models is very high. In fact, Rim et al. have already shown differences in prediction rates of CVD risk factors for their fundus photography DL algorithm when applied to samples of different patient demographics [197].

Additionally, the potential for systemic failure caused by continuous learning AI models adjusting algorithms based on errors is a limitation for scalability as well. If left unchecked, continuous learning models may become dysfunctional after making too many algorithmic adjustments based off only a few errors. Regulatory action would be necessary to control this systemic error, including regular quality control tests, and to avoid AI model dysfunction.

Therefore, prior to wide-scale integration into clinical practice, there are numerous preliminary steps that can be completed to address the aforementioned ethical considerations. Federated learning governance allows for the collaborative training of AI models across multiple institutions by leveraging datasets from these different centers [198,199,200]. Creation of these learning networks not only ensures data privacy as patient/user data remains local, but it also inherently promotes standardization of imaging protocols as AI models are exposed to a greater collective of expertise and training resources [198,199,200]. This method of data acquisition not only accounts for security concerns, but the inherent heterogeneity of the collected data bolsters AI model scalability [198,199,200]. Independent auditing can be conducted to further evaluate the safety and efficacy of these novel algorithms. Examples of these checkpoints include external validation by testing datasets not used in the model’s development, the requirement of complete transparency and reporting of AI model and development processes, and continuous post-deployment monitoring.

AI models need to undergo validation with rigorous clinical trials, not only establishing their value but also comparing their true clinical benefit in comparison to the current gold standards [Table 5]. These trials are needed to evaluate whether there is value in adding these models to our clinical practice, as it is imperative to determine whether the cost of implementation, whether it is time, financially, or in data procurement, does not outweigh the benefits. When comparing in-house or minute-clinic retinal biomarker assessment with other manners of assessing CVD status, retinal modalities present numerous avenues through which significant cost savings can be achieved. These range from reduced infrastructure, training requirements, and operational costs to streamlined integration into preclinical and clinical workspaces and high repeatability. Supplementation of traditional CVD status assessment with retinal biomarker assessments represents a potential pathway through which broad population screening can consistently be achieved both inside and outside the clinical setting.

## 9. Conclusions

While the eye has long been regarded as a window to the central nervous system, its microvasculature is increasingly proving itself to be a window into the entire cardiovascular system. Given the associations between the cardiac and ocular vascular microenvironment, it seems natural that the eye may provide cardiologists and other non-ophthalmologic clinicians with a noninvasive view into their patients’ microvascular health. As noted throughout this review, there are many ocular biomarkers and pathologies that provide insight into the cardiovascular health of patients. Advancements in AI postprocessing and image acquisition only further the potential of the field of cardiac-oculomics and its ability to play a crucial role in the prevention, diagnosis, and management of patients’ CVD.

While a variety of challenges exist towards the implementation of ophthalmic imaging into the cardiovascular clinical environment, including variability in imaging, AI-related challenges, and health data privacy, these obstacles are not insurmountable. Tangible steps can be taken to improve all aspects of this process, including automated imaging, biomarker identification, risk stratification, and clinical recommendation. With this information in hand, collaboration between cardiologists and ophthalmologists is the optimal way forward to build clinically relevant solutions to optimize patient outcomes for personalized prevention and treatment of CVD.

## Figures and Tables

**Table 1:** Color fundus photography biomarkers and associated cardiovascular diseases.

Biomarker	Significance	Associated Cardiovascular Disease
**Arteriovenous Nicking**	Indentation and compression of a venule when an arteriole crosses over it	Hypertension
**Retinal Vessel Caliber**	The cross-sectional width of retinal arterioles and venules	Hypertension
**A/V Ratio**	A ratio between the relative caliber of retinal arteries in comparison to retinal veins	Hypertension
**Central Retinal Arteriole Equivalent**	The average diameter of the arterioles within the retina	Hypertension
**Central Retinal Venular Equivalent**	The average diameter of the venules within the retina	Hypertension
**Retinal Arteriolar and Venule Diameter**	The diameter of the column of blood cells present within the lumen of a vessel that indicates widening or narrowing at the time of capture	Myocardial infarction/coronary artery disease Heart failure
**Retinal Hemorrhage**	Bleeding that occurs within the retina	Hypertension Myocardial infarction/coronary artery disease
**Cotton Wool Spots**	Discrete white lesions that lie within the retinal nerve fiber layer	Myocardial infarction/coronary artery disease Cerebral infarction Heart failure

**Table 2:** Optical coherence tomography biomarkers and associated cardiovascular diseases.

Biomarker	Significance	Associated Cardiovascular Disease
**Retinal Nerve Fiber Layer Thickness**	A measurement of the thickness of the layer of the retina consisting of the axons of the retinal ganglion cells	Myocardial infarction/coronary artery disease Cerebral Infarction Heart failure
**Paracentral Acute Middle Maculopathy Lesions**	Retinal abnormalities occurring in the inner nuclear layer of the retina that manifest as hyperreflective bands	Hypertension Carotid artery disease
**Retinal Ischemic Perivascular Lesions**	Focal areas of ischemic thinning occurring along the retinal blood vessels in the inner retina	Hypertension Myocardial infarction/coronary artery disease Cerebral infarction Carotid artery disease Atrial fibrillation
**Subretinal Drusenoid Deposits**	Extracellular protein deposits located above the retinal pigment epithelium but below the photoreceptor layer	Hypertension Myocardial infarction/coronary artery disease Carotid artery disease Valvular heart disease Angina
**Subfoveal Choroidal Thickness**	The thickness of the vasculature bed between the retinal pigment epithelium and the sclera	Myocardial infarction/coronary artery disease Heart failure

**Table 3:** Optical coherence tomography angiography biomarkers and associated cardiovascular diseases.

Biomarker.	Significance	Associated Cardiovascular Disease
**Foveal Avascular Zone**	The central area of the retina that is devoid of blood vessels from which several measurements can be derived, including the area, circularity, and the length of the capillary ring surrounding the region	Hypertension Carotid artery disease
**Vessel Area Density**	The ratio of the retinal area covered by binarized blood vessels to the total retinal area	Hypertension Myocardial infarction/coronary artery disease Cerebral infarction Carotid artery disease
**Vessel Length Density**	The ratio of the total length of 1-pixel-wide skeletonized blood vessels to the total retinal area	Hypertension Carotid artery disease Valvular heart disease

**Table 4:** Challenges and limitations to implementing cardiac-oculomics in clinical practice.

	Challenge/Limitations	Impact	Future Directions/Proposed Solutions
**Technical Challenges (Retinal Imaging)**	Variability in retinal imaging techniques (e.g., differences in hardware specifications, software algorithms, and operator expertise)	1. Differences in size, area, and resolution of images obtained 2. Significant variance in image interpretation 3. Difficulty in constructing an algorithm to apply to a broader patient population	1. Standardization of image acquisition protocols to aid in consistency of size, area, and resolution 2. Regular calibration of imaging devices and cross-validation of images 3. Implementation of training certification programs and qualifying examinations to ensure a certain level of expertise in image acquisition and interpretation 4. Utilization of a diverse patient cohort when testing new imaging protocols to better prepare for patient characteristics (anatomy, unexpected movement) deviating from “normal” in a routine clinical setting
**AI-Related Challenges**	Black Box Algorithms—inability for humans to interpret how an algorithm reached a certain conclusion	1. Lack of model transparency 2. Lack of trust by providers in AI-based machinery 3. Perpetuation of errors without correction	1. Incorporation of explainable AI to build provider trust, as this addition would enable AI models to explain the reasoning behind a certain conclusion 2. Implementation of class activation mapping to allow for transparent and consistent biomarker and feature identification
	Attribution of responsibility	1. Legal and ethical dilemmas over whether healthcare providers should be held responsible for utilizing an algorithm’s incorrect result	1. Extensive validation of AI models through clinical trials before implementation in a broader patient population
	Bias in AI models	1. Disproportionate misdiagnosis of conditions amongst disadvantaged groups 2. Widening of existing inequality in healthcare if an algorithm infiltrated with bias is implemented in standard clinical practice	1. Representation of an extensive variety of patient demographics in AI training sets 2. Train highly specific models to allow for personalized risk identification catered to sub-populations
	Continuous Learning	1. Unsupervised algorithms can become dysfunctional after making multiple adjustments based on few errors 2. Potential for harm to patients through compromised clinical decision-making	1. Regular quality control tests to avoid dysfunction of AI models
	Health Data Privacy	1. Potential for patient health data misuse 2. Infringement of patient privacy	1. Use of privacy-preserving technologies such as federated learning and blockchain 2. Developing standardized models for collaboration between health care entities and AI companies to maximize patient safety and efficient use of data for the benefit of future patients

**Table 5:** Proposed future exploration and areas of study.

Further Areas of Study	Potential Research Questions/Proposed Future Studies
**Retinal Artery/Vein Occlusion**	What is the efficacy of incorporating further cardiovascular evaluation and pharmacological intervention (e.g., statins) after diagnosis of an RAO or RVO for the prevention of future cardiovascular events? Given the association CRAO has with atrial fibrillation, is there an association between CRAO and retinal biomarkers typically seen in the event of atrial fibrillation (e.g., RIPLs)?
**Hypertension**	Is there a difference in the distribution and number of RIPLs associated with different stages of hypertension? Perform a meta-analysis to evaluate the relationship between hypertension and SCP/DCP VAD and VLD, using existing data in the literature.
**Coronary Artery Disease, Myocardial Infarctions, and Atherosclerotic Disease**	Assess what role multimodal retinal imaging could play in the emergency room workflow for evaluation of patients with chest pain. Could retinal imaging assist in determining the root cause and subsequent treatment (STEMI, NSTEMI, stable angina, etc.)? Are any alterations in the foveal avascular zone observed with these disease states?
**Valvular Heart Disease**	What specific findings on CFP, OCT, and OCTA are correlated with increased prevalence of aortic valve regurgitation?
**Heart Failure**	Is there an increase in the presence of RIPLs and SDDs in the HF population, and if so, after administration of GDMT, are there any changes in the number and severity of retinal biomarkers observed?
**Cerebral Infarctions**	Are there differences in retinal manifestations that can help distinguish between a transient ischemic attack and a stroke?
**Atrial Fibrillation**	What other retinal biomarkers of ischemia observed on OCT/OCTA are correlated with atrial fibrillation?
**Cardiovascular Disease Risk Scores**	Does incorporation of CFP-retinal biomarkers into existing cardiovascular prediction algorithms such as the ASCVD risk calculator and the PREVENT risk calculator aid in capturing a greater number of patients earlier on in their disease course for more effective treatment? Assess the clinical utility of using multimodal retinal imaging in the primary care setting to see how it further assists CVD risk assessments and alters treatment initiation, disease monitoring, and treatment efficacy.
**Incorporation of retinal biomarkers into standard clinical workflow**	Can retinal imaging be successfully incorporated in primary care offices in underserved and rural areas as an alternative strategy to more costly screening methods such as CAC? What is the cost-effectiveness analysis comparison between multimodal retinal imaging and standard methods for cardiovascular disease evaluation?

## Data Availability

No data was procured for this review article; therefore, no data is available to share with the readership.

## Abbreviations

The following abbreviations are used in this manuscript:

ADHF	Acute Decompensated Heart Failure
AHA	American Heart Association
AI	Artificial Intelligence
AMD	Age-related Macular Degeneration
AMN	Acute Macular Neuroretinopathy
AR	Aortic Regurgitation
ASCVD	Atherosclerotic Cardiovascular Disease
AV	Arteriovenous
BM	Bruch’s Membrane
BMI	Body Mass Index
BRAO	Branch Retinal Artery Occlusion
BRVO	Branch Retinal Vein Occlusion
CAC	Coronary Artery Calcium
CAD	Coronary Artery Disease
CAS	Carotid Artery Stenosis
CC	Choriocapillaris
CFP	Color Fundus Imaging
CHF	Congestive Heart Failure
CNN	Convolutional Neural Network
CRAE	Central Retinal Arteriolar Equivalent
CRAO	Central Retinal Artery Occlusion
CRVE	Central Retinal Venous Equivalent
CRVO	Central Retinal Vein Occlusion
CVD	Cardiovascular Disease
CWS	Cotton Wool Spot
DCP	Deep Capillary Plexus
DL	Deep Learning
DR	Diabetic Retinopathy
EF	Ejection Fraction
FA	Fluorescein Angiography
FAZ	Foveal Avascular Zone
GRACE	Global Registry of Acute Coronary Events
HF	Heart Failure
HFpEF	Heart Failure with Preserved Ejection Fraction
HFrEF	Heart Failure with Reduced Ejection Fraction
ICA	Internal Carotid Artery
ICGA	Indocyanine Green Angiography
ICP	Intermediate Capillary Plexus
INL	Inner Nuclear Layer
IV	Intravenous
LCX	Left Circumflex Coronary Artery
LMCA	Left Main Coronary Artery
MI	Myocardial Infarction
MRI	Magnetic Resonance Imaging
OCT	Optical Coherence Tomography
OCTA	Optical Coherence Tomography Angiography
ONL	Outer Nuclear Layer
OPL	Outer Plexiform Layer
PAMM	Paracentral Acute Middle Maculopathy
PET/CT	Positron Emission Tomography/Computed Tomography
PREVENT	Predicting Risk of Cardiovascular Disease Events
RAO	Retinal Artery Occlusion
RCA	Right Coronary Artery
REACH	Reduction of Atherothrombosis for Continued Health
RIPL	Retinal Ischemic Perivascular Lesion
RNFL	Retinal Nerve Fiber Layer
RPCP	Radical Peripapillary Capillary Plexus
RPE	Retinal Pigment Epithelium
RVA	Retinal Vessel Analysis
RVO	Retinal Vein Occlusion
SCP	Superficial Capillary Plexus
SD-OCT	Spectral Domain-Optical Coherence Tomography
SDD	Subretinal Drusenoid Deposit
SFCT	Sub-foveal Choroidal Thickness
SRVA	Static Retinal Vessel Analysis
SS-OCT	Swept Source-Optical Coherence Tomography
TIMI	Thrombolysis of Myocardial Infarction
VAD	Vessel Area Density
VLD	Vessel Length Density
